# Multidisciplinary ocular and periocular cancers meetings: implementation in a tertiary referral center and analysis over a 12-months period

**DOI:** 10.1186/s12886-022-02694-3

**Published:** 2022-12-19

**Authors:** Gustavo Savino, Fabrizio Piccinni, Monica Maria Pagliara, Maria Grazia Sammarco, Carmela Grazia Caputo, Alessandro Moro, Giorgio Barbera, Luca Tagliaferri, Bruno Fionda, Giovanni Schinzari, Ernesto Rossi, Luca Zagaria, Ketty Peris, Alessandro Di Stefani, Teresa Musarra, Luca Ausili Cefaro, Matia Martucci, Maria Antonietta Blasi

**Affiliations:** 1 Ocular Oncology Unit, Fondazione Policlinico Universitario A. Gemelli IRCCS, Rome, Italy; 2 Università Cattolica del Sacro Cuore, UCSC, Rome, Italy; 3 Maxillo-Facial Surgery Unit, Fondazione Policlinico Universitario A. Gemelli IRCCS, Rome, Italy; 4 UOC Radioterapia Oncologica, Dipartimento di Diagnostica per Immagini, Radioterapia Oncologica ed Ematologia, Fondazione Policlinico Universitario A. Gemelli IRCCS, Rome, Italy; 5 Medical Oncology Unit, Fondazione Policlinico Universitario A. Gemelli IRCCS, Rome, Italy; 6 Nuclear Medicine Unit, Fondazione Policlinico Universitario A. Gemelli IRCCS, Rome, Italy; 7 Dermatology Unit, Fondazione Policlinico Universitario A. Gemelli IRCCS, Rome, Italy; 8 Division of Anatomic Pathology and Histology, Fondazione Policlinico Universitario A. Gemelli IRCCS, Rome, Italy; 9 UOSD Neuroradiologia Diagnostica, Dipartimento di Diagnostica per Immagini, Radioterapia Oncologica ed Ematologia, Fondazione Policlinico Universitario A. Gemelli IRCCS, Rome, Italy

**Keywords:** Ophthalmology, Oncology, Ocular oncology, Ocular oncology multidisciplinary tumor board, Multidisciplinary team, Ocular MDTB

## Abstract

**Purpose:**

The complexity of multimodal approaches in cancer management has lately led to the establishment of multidisciplinary tumor boards (MDTBs) to define targeted, patient-centered treatment strategies. However, few data are available regarding the application of this approach in Ocular Oncology. Hereby, the Authors analyze the implementation and outcomes of a trained MDTB in a tertiary ocular oncology referral center.

**Methods:**

A retrospective descriptive analysis of MDTB meetings discussing patients with ocular and periocular cancers, over a 12-months period, was carried out. Data were grouped by main site involved, topics discussed and final clinical decisions therefore taken. Meetings were held by a constant ‘Core team’ or – when required – by a broader ‘Extended team’.

**Results:**

During the observational period 86 cases were discussed. In 27 patients ocular surface tissues were involved (31%), in 25 patients orbital tissues (29%), in 22 patients eyelids (26%), and in 12 patients intraocular tissues (14%). In 13 cases (15%) naïve or referred new patients, in 34 cases (40%) imaging or histopathologic reports and in 39 cases (45%) treatment plans were discussed. Regarding final decisions, a treatment plan was scheduled in 47 cases (55%) and a diagnostic ascertainment was required in 27 patients (31%); locally advanced and/or systemic diseases were referred or teamed up with other specialists in 12 cases (14%).

**Conclusions:**

Ocular Oncology multidisciplinary team, by sharing expertise of different specialists, ensures a comprehensive evaluation of patients improving the accuracy of diagnosis and staging upon which planning a proper treatment. Further studies are needed to assess if this approach may also improve the outcomes and prognosis of patients.

## Introduction

Cancer management and treatment involves a complexity of multimodal strategies and approaches that require a detailed background in different medical specialties.

The heterogeneity of the disease, as well as the multimodal treatments available (such as surgery, radiation, and chemotherapy), have raised the request for the establishment of multi-disciplinary teams (MDTs) in different specialties for a holistic evaluation of patients to provide an integrated and individually tailored treatment plan.

Multidisciplinary Team Management (MDTM) refers to the method of clinical evaluation and treatment planning that involves the participation of physicians enrolled in different, yet related, medical specialties. The main way by which MDTM works is by holding regular meetings, where clinical cases of cancer patients are discussed, and each patient receives a personalized diagnosis and treatment plan. Both new cases and follow-up are discussed in each meeting [1].

Little evidence is available regarding the application of this approach in Ocular Oncology and aim of this manuscript is to describe the implementation of this method in the management of patients with ocular and periocular cancers. An Ocular Oncology Multi-Disciplinary Tumor Board (MDTB) has been trained in the last few years at our tertiary referral center and the Authors review a 12-months activity. Team composition, different sites affected, main topics discussed, and implementation of final outcomes are analyzed.

Results are both presented in an overall view and divided in subgroups depending on the main site affected (orbit, eyelids, ocular surface and intraocular tissues).

## Materials and methods

The Authors analyze the activity of the MDTB of the Department of Ocular Oncology of *“Fondazione Policlinico Universitario A. Gemelli IRCSS”* of Rome between October 2020 and October 2021. Meetings were held monthly at our tertiary referral center and a retrospective descriptive analysis of cases discussed was carried out. All challenging cases requiring a multidisciplinary opinion on clinical and/or histopathological diagnosis, staging or restaging, or treatment planning were discussed.

Data were collected as (Table 1): a) main site involved; b) main points discussed during meeting; c) final outcomes (clinical decision taken after meetings).

**Table 1:** Analysis and characterization of multi-disciplinary tumor board discussions based on sites involved, points discussed and final outcomes

MDTB Analysis	
**a) Main sites involved**	- A) Orbit - B) Ocular Surface - C) Eyelids - D) Intraocular tissues
**b) Main points discussed**	- First presentation *Management of a first clinical presentation of a suspected or ascertained ocular or periocular cancer* - Imaging or Histopathological evaluation *Shared team discussion of radiologic imaging (eg: MRI, CT) or histopathological reports of patients with a diagnosis of suspected or ascertained ocular or periocular tumor* - Treatment planning *Discussion of first treatments proposals, or adjustments of previously established treatments, or further treatment proposal in patients with relapsing cancer (eg: local recurrence or therapy-refractory tumor).*
**c) Meeting outcome**	- Diagnostic *Incisional biopsy or further imaging (eg: CT, RMI, PET) is requested to establish diagnosis or to stage/restage already diagnosed cancers* - Therapeutic *A medical and/or surgical treatment is defined (either as a first proposal or a further treatment in already-treated patients with relapsing features), or a previous planned treatment is modified (or, less commonly, watchful waiting is proposed)* - Referral *Cases referred to other specialists (OMFS/ENTs, Dermatologist, Oncologists, Pathologists)*

Over this 12-months period, 86 cases were discussed during 12 total, monthly-held meetings; 71 patients were discussed only once (and a shared team decision was achieved), whereas 15 patients have been discussed 2 or more times in different meetings. A dedicated platform for data collection was used to retrieve anonymized data retrospectively [2].

### MDTB composition

Even if a tumor board is the ideal setting to discuss complex clinical cases, no guidelines are currently available on which specialists should be included in an ocular oncology MDTB. Moreover, it was reported that physicians’ decisions can be affected by several factors, including physicians’ specialty and experience [3]. Guidelines and legal requirements are difficult to identify and access even for Head and Neck Cancer tumor boards, where this approach has been fairly implemented; suggested composition of the Core Team varies across countries, with guidelines supporting both smaller and larger approaches [4].

Our board included a “Core Team”, consisting of Ophthalmologists and Ocular Oncologists, Medical Oncologists, Radiation Oncologists, Radiologists, Pathologists, Maxillofacial Surgeons (OMFS) and Otolaryngologists (ENTs). A Clinical Trial Coordinator and a MDTB Meeting Coordinator were also enrolled (Table 2). Core Team members have always been present during meetings; an Extended Team of specialists (such as Dermatologists and Nuclear Physicians) has been involved where necessary.

**Table 2:** Composition of our Ocular Oncology Multi-Disciplinary Tumor Board

Ocular Oncology MDTB	
**Core Team**	
- Ophthalmologists & Ocular Oncologists	
- Medical Oncologists	
- Radiation Oncologists	
- Otolaringologists (ENTs) & Maxillofacial Surgeons (OMFS)	
- Radiologists	
- Pathologist	
- Clinical Trial Coordinator	
- MDTB meeting coordinator	
**Extended Team**	
- Dermatologist	
- Nuclear Physician	

## Results

### Overall view

During the 12 months study period, 86 patients with suspected or confirmed ocular or periocular cancers were discussed out of a total of 2320 patients (360 first and 1920 follow-up visits) treated at our Ocular Oncology Unit (3.7%). Mean age of patients was 67 +/− 17 years old. 53% of patients (46/86) were males and 47% (40/86) females. All patients were Caucasian. Tumors occurred on the right side in 48% of patients and on the left side in 52%.

Mean time of discussion was 13 min for each case. Mean time from presentation of patients at our Ocular Oncology Unit to MDTB discussion was 13 days (range: 2-27 days). Mean time to application of recommendations (diagnostic, therapeutic or referral decision) was: 27 days (range: 5-34 days) for diagnostic ascertainments, 10 days (range: 4-19 days) to perform medical or surgical treatments and 13 days (range: 3-23 days) to get a referral to other specialists.

**Fig. 1:**
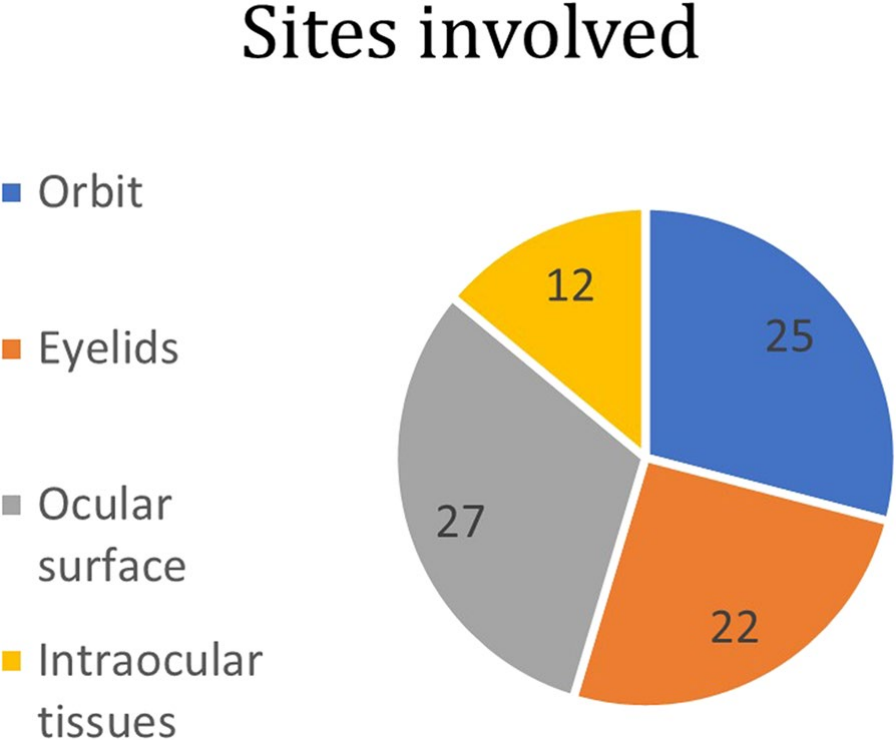
Cases discussed, grouped by main ocular site involved

Main **sites** involved included (Fig. 1): orbit (25 out of 86 patients, 29%), eyelids (22 out of 86 cases, 26%), ocular surface (27 out of 86 patients, 31%) and intraocular tissues (12 out of 86 cases, 14%).

**Fig. 2:**
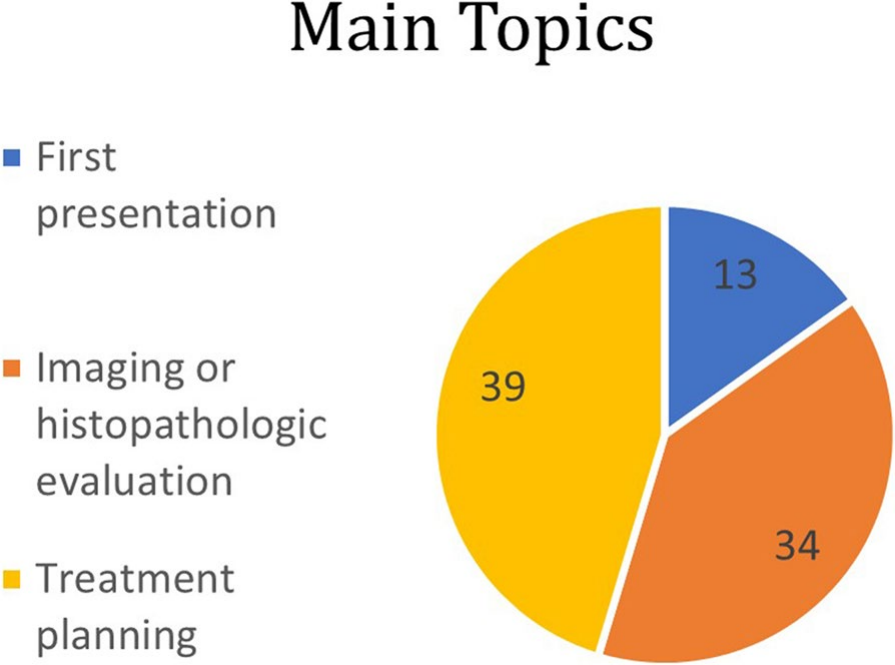
Main topics discussed – Overall

**Fig. 3:**
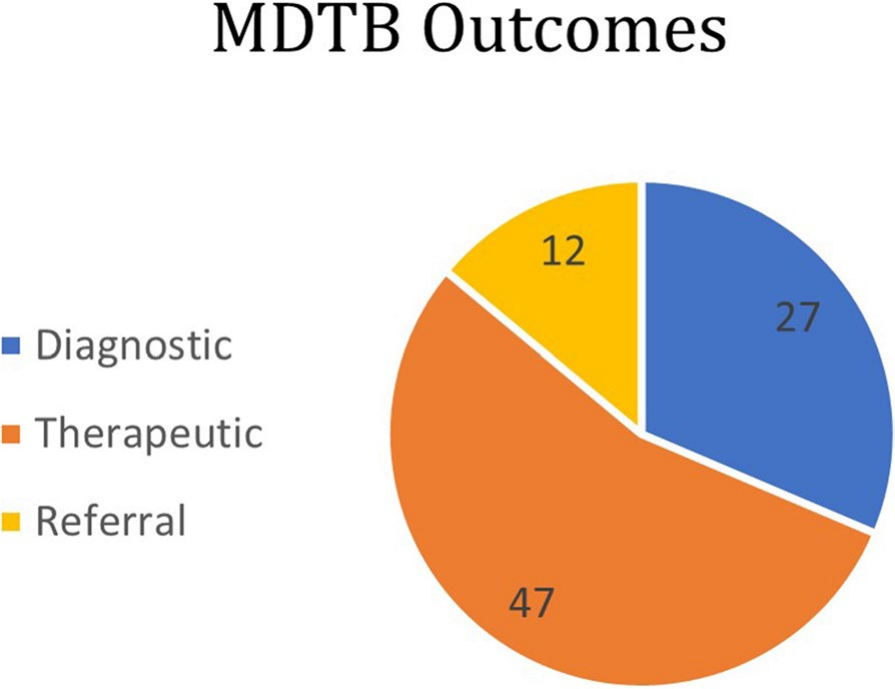
Final decisions taken - Overall

Main **topics** discussed (Fig. 2) included an evaluation of a first clinical presentation of suspected ophthalmic cancer in 13 out of 86 (15%): 1 case each in the orbit group and intraocular tissue group, 5 cases in the eyelids group and 6 cases in ocular surface group. An evaluation of imaging acquisitions (mainly Magnetic Resonance Imaging/MRI or Computed Tomography/CT images) and/or histopathological reports suggestive of ophthalmic cancers was discussed in 34/86 cases (40%), 18 cases in orbital group, 14 in eyelids group and 1 case each in ocular surface and intraocular tissues groups). A planning of medical or surgical treatment was inquired in 39 out of 86 cases (45%), some of which (13 out of 39) in patients with relapsing or therapy-refractory tumors. Six treatment plans were discussed in orbital group, 3 in eyelids group, 20 in ocular surface group and 10 in intraocular tissue group. Final **outcomes** (Fig. 3) after meeting discussions were: treatment planning in 47 cases (55%), diagnostic ascertainment in 27 cases (31%) and referring requirements to other specialists in 12 cases (14%).

### Orbit

#### Cases discussed

Orbital tumors included 25 of 86 patients discussed (29%). In 11 out of 25 cases, orbital non-vascular tumors (mainly comprising orbital lesions detected by RMN imaging) were discussed. Discussions also included cavernous venous malformations in 3 cases, lacrimal gland tumors in 4 cases (including two adenocarcinomas) and a new-onset proptosis of unknown origin in 2 cases. In 5 out of 25 cases other less common orbital disorders (such as orbital cysts, an optic nerve glioma, a myofibroblastic inflammatory tumor and a solitary orbital fibrous tumor) were discussed (Table 3).

**Table 3:** Orbital cases presented (grouped by clinical presentations, topics discussed and meetings’ outcomes)

Orbit		
Clinical presentation *[Cases number]*	Topics discussed *(cases number)*	MDTB Outcomes
Non-Vascular Tumors *(presenting as orbital lesions) [11/25]*	Evaluation of Imaging *(10 cases)*	Diagnostic *Incisional biopsy required (8 cases)*
		Therapeutic *Excisional biopsy (2 cases)*
	Evaluation of histopathological report *(1 case)*	Referral *→ to Pathologist (second opinion)*^*a*^
Cavernous Venous Malformations *[3/25]*	Treatment planning *(3 cases)*	Therapeutic *Excisional biopsy (3 cases)*
Lacrimal Gland Tumors *[4/25]*	Evaluation of Imaging *(4 cases)*	Diagnostic *Incisional biopsy required (1 case)*
		Therapeutic *Watchful waiting (1 case) or exenteration orbitae (2 cases)*
Undetermined Proptosis *[2/25]*	Discussion of first presentation *(1 case)*	Diagnostic *MRI required*
	Evaluation of Imaging *(1 case)*	Diagnostic *Incisional biopsy required*
*Miscellaneous diseases*		
Cyst/Orbital Granulomas *[2 cases]*	Treatment planning *(2 cases)*	Therapeutic *Excisional biopsy (1 case) or watchful waiting (1 case)*
Optic Nerve Glioma *[one case]*	Evaluation of imaging	Therapeutic *Watchful waiting*
Myofibroblastic Inflammatory Tumor *[one case]*	Treatment planning	Therapeutic *Excisional biopsy*
Solitary Fibrous Tumor *[one case]*	Evaluation of histopathological report	Therapeutic *Watchful waiting*

^a^Second opinion confirms an orbital melanoma in place of a previously diagnosed blue nevus, de facto changing diagnosis

#### Main points

**Fig. 4:**
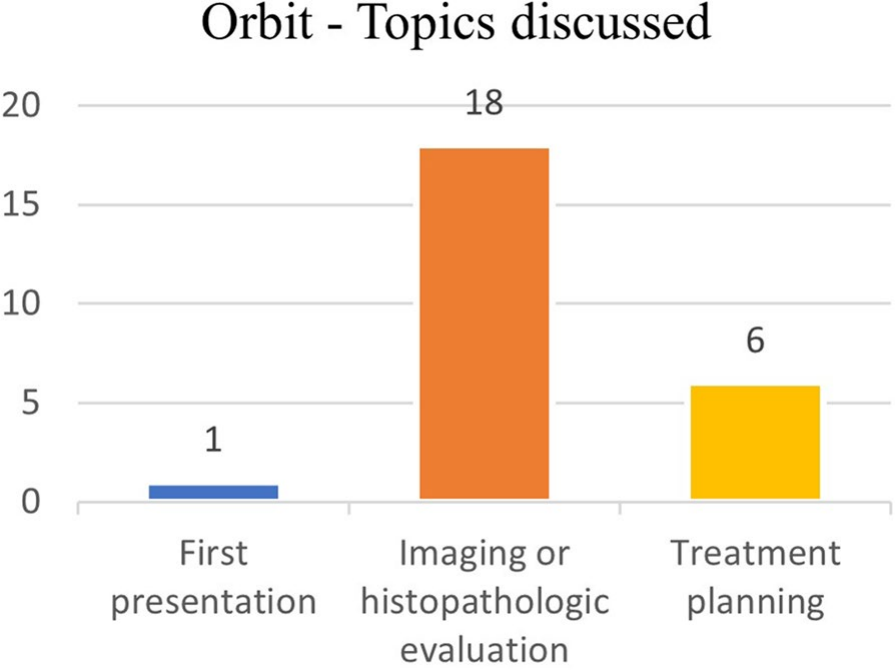
Orbit – Main topics discussed

In 18 out of 25 cases (72%), histopathological biopsy reports or imaging scans (MRI and/or PET-CT and/or CT, 17 out of 18 patients) were analyzed and discussed. In 6 out of 25 (24%) patients treatment planning was discussed. Only one case regarded a de novo clinical presentation (Fig. 4).

#### MDTB outcomes

**Fig. 5:**
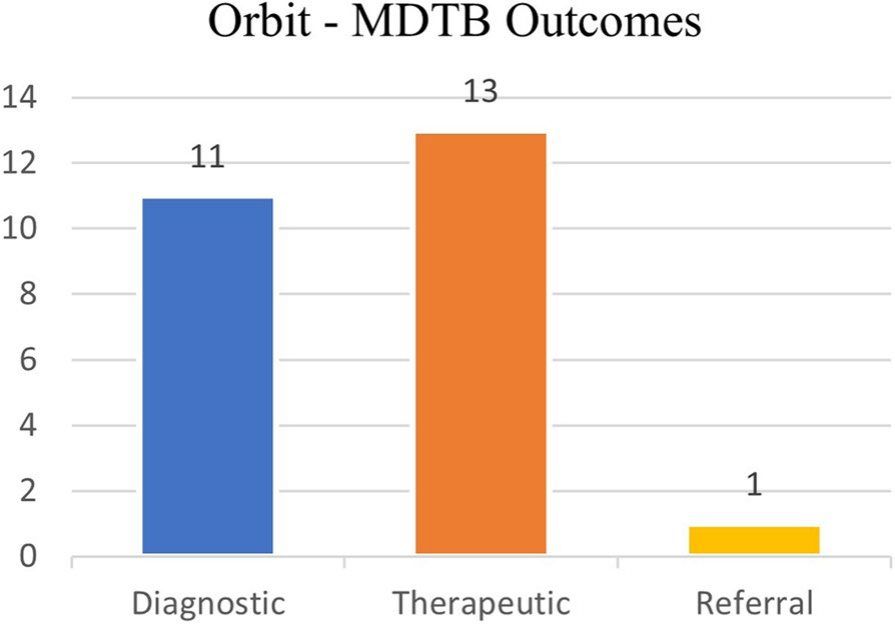
Orbit – Final decisions taken

A further diagnostic assessment was required in 11/25 patients (44%), mainly scheduling incisional biopsies (10 cases). A therapeutic decision (watchful waiting or surgical treatment, mainly consisting of excisional biopsies) was taken in 13 out 25 cases (52%). In one case, a referral to our Pathologists for a second opinion on a doubtful histopathological specimen was required (Fig. 5).

### Eyelids

#### Cases discussed

Twenty-two out of 86 cases discussed (26%) presented eyelids tumors (Table 4) including: six basal cell carcinomas, four sebaceous carcinomas, two squamous cell carcinomas, two Merkel carcinomas, a porocarcinoma, a Diffuse Large B-Cell Lymphoma (DLCBL) and an eyelid melanoma; five cases pertained suspected eyelid tumors, presenting as new-onset eyelid lesions or swelling.

**Table 4:** Eyelids cases presented (grouped by clinical presentations, topics discussed and meetings’ outcomes)

Eyelids		
Clinical presentation *[Cases number]*	Topics discussed *(cases number)*	MDTB Outcomes
Basal Cell Carcinomas *[6/22]*	Evaluation of imaging *(one case)*	Diagnostic *Incisional biopsy required*
	Evaluation of histopathological reports *(4 cases)*	Referral *→ to Dermatologists (for Sonidegib treatment, 2 cases)*
		Therapeutic *Watchful waiting (2 cases)*
	Treatment planning *(relapsing cancer, one case)*	Referral *→ to Dermatologists (for Vismodegib treatment, one case)*
Sebaceous Carcinomas *[4/22]*	Evaluation of histopathological report *(4 cases)*	Diagnostic *CT + sentinel lymph-node biopsy required for tumor staging (4 cases)*
Squamous Cell Carcinomas *[2/22]*	Treatment planning *(relapsing cancer, one case)*	Therapeutic *Excisional biopsy*
	Evaluation of histopathological report *(one case)*	Diagnostic *CT + sentinel lymph-node biopsy required for tumor staging*
Eyelid Undiagnosed Lesions *[5/22]*	Discussion of first presentations *(5 cases)*	Diagnostic *Incisional biopsy (2 cases) or TC (1 case) required*
		Therapeutic *Excisional biopsy (2 cases)*
Merkel Carcinomas *[2/22]*	Treatment planning *(relapsing cancer, one case)*	Referral *→ to Oncologists (systemic metastasis detected)*
	Evaluation of histopathological report *(one case)*	Diagnostic *CT + sentinel lymph-node biopsy required for tumor staging*
Porocarcinoma *[one case]*	Evaluation of histopathological report	Diagnostic *CT + sentinel lymph-node biopsy required for tumor staging*
DLBCL *[one case]*	Evaluation of histopathological report	Referral *→ to Hematologists*
Eyelid Melanoma *[one case]*	Evaluation of histopathological report	Referral *→ to Dermatologists/Oncologists*

#### Main points

**Fig. 6:**
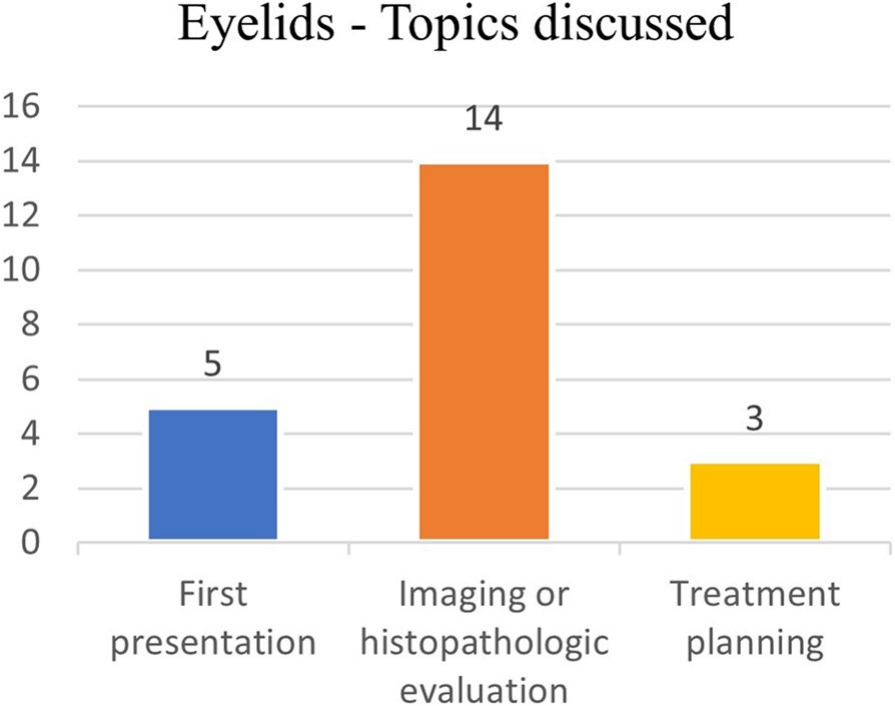
Eyelids – Main topics discussed

In 14 out of 22 cases (63%), imaging scans or histopathologic reports (13 out of 14 patients) were discussed. Treatment planning (particularly pertaining previously treated, yet relapsing tumors) was inquired in 3 cases (14%), and in 5 cases (23%) a first clinical presentation of undiagnosed suspected eyelid tumors was discussed (Fig. 6).

#### MDTB outcomes

**Fig. 7:**
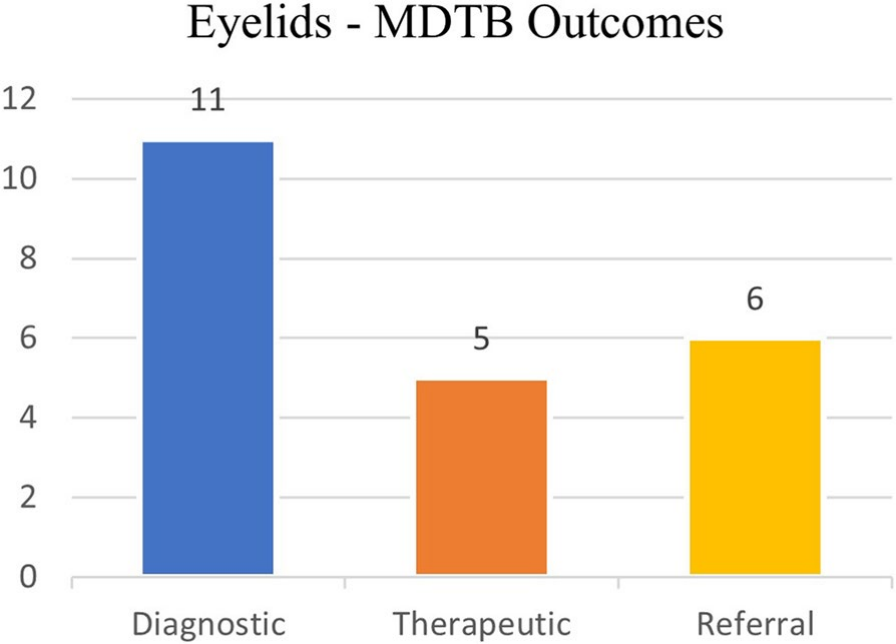
Eyelids – Final decisions taken

A further diagnostic ascertainment was required in 11 out of 22 patients (50%) through incisional biopsies, MRI/CT examination or tumor staging (through CT imaging + sentinel lymph-node biopsy). Patients were referred to other specialists (mainly Dermatologists or Oncologists) in 6 out of 22 cases (27%). A therapeutic decision (watchful waiting or excisional biopsy) was taken in 5 out of 22 cases (23%) (Fig. 7).

### Ocular surface

#### Cases discussed

Twenty-seven out of 86 cases (31%) discussed ocular surface lesions (Table 5), mostly consisting of OSSNs (Ocular Surface Squamous Neoplasia, 14 out of 27) or conjunctival melanomas (9 out of 27); four cases pertained an undiagnosed first clinical presentation of pigmented or salmon-pink conjunctival lesions.

**Table 5:** Cases presented involving ocular surface (grouped by clinical presentations, topics discussed and meetings’ outcomes)

Ocular Surface		
Clinical presentation *[Cases number]*	Topics discussed *(cases number)*	MDTB Outcomes
Ocular Surface Squamous Neoplasia (OSSN) *[14/27]*	Discussion of first presentation *(one case)*	Diagnostic *Incisional biopsy*
	Treatment planning *(13 cases, 7 of which in previously treated patients with relapsing disease)*	Therapeutic *Excisional biopsy and/or local brachytherapy and/or topical Mitomycin C (12 cases)*
		Referral *→ to Oncologists (one case)*
Conjunctival Melanoma *[9/27]*	Treatment planning *(7 cases, 3 of which in previously treated patients with relapsing disease)*	Therapeutic *Brachytherapy (6 cases)*
		Diagnostic *CT + sentinel lymph-node biopsy required for tumor staging (one case)*
	Evaluation of histopathological report *(one case)*	Referral *→ to Pathologist (second opinion)*^*a*^
	Discussion of first presentation *(one case)*	Therapeutic *Excisional biopsy*
Pigmented Conjunctival Lesion *[3/27]*	Discussion of first presentation *(3 cases)*	Therapeutic *Excisional biopsy (3 cases)*
Salmon-pink Conjunctival Lesion *[1/27]*	Discussion of first presentation *(one case)*	Diagnostic *Incisional biopsy required*

^a^Second opinion confirms a diagnosis of conjunctival melanoma in place of a previously diagnosed lentigo maligna, de facto changing diagnosis

#### Main points

**Fig. 8:**
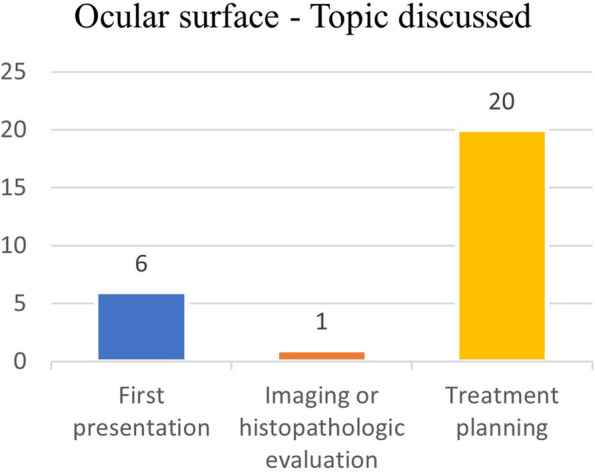
Ocular surface – Main topic discussed

Treatment planning of naïve patients (10 cases) or patients with relapsing disease (10 cases) was discussed in 20 out of 27 cases (74%); in 6 cases (22%) a new clinical presentation was inquired and in one case a histopathologic report was evaluated (Fig. 8).

#### MDTB outcomes

**Fig. 9:**
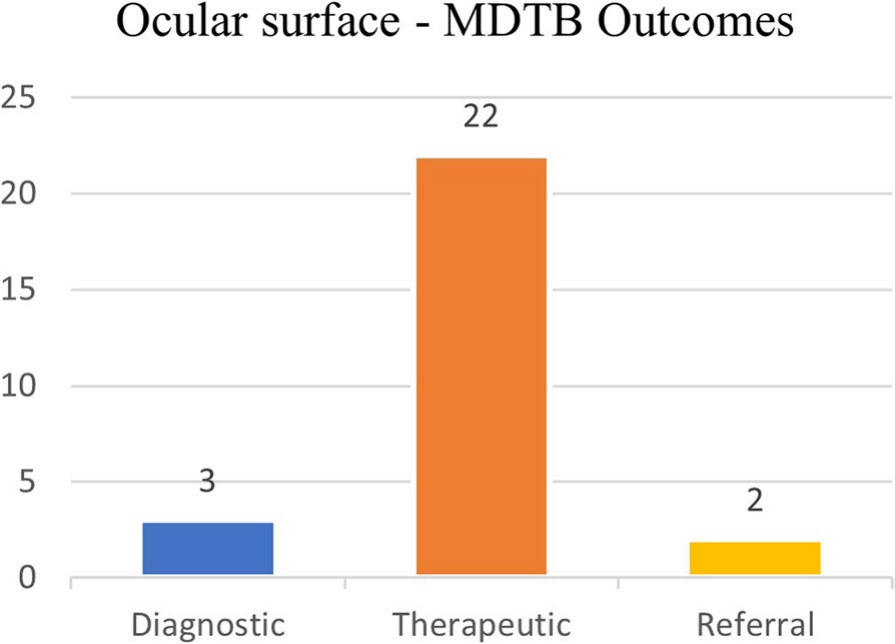
Ocular surface – Final decisions taken

A further diagnostic examination was requested in 3 out of 27 patients (11%) through incisional biopsies (2 cases) or CT + sentinel node biopsy for tumor staging. A therapeutic surgical (through excisional biopsies execution) or radiotherapic (via brachytherapy sessions) treatment was recommended in 22 out of 27 cases (82%). In 2 out of 27 cases (7%), patients were referred to other specialists (Fig. 9).

### Intraocular tissue

#### Cases discussed

Twelve out of 86 cases (14%) presented intraocular tissues tumors (Table 6). Ten patients were affected by choroidal melanomas, five of which had already been previously enucleated. Two cases were in relation to suspected choroidal metastasis in patients affected by known malignancies.

**Table 6:** Cases presented involving intraocular tissues (grouped by clinical presentations, topics discussed and meetings’ outcomes)

Intraocular Tissues		
Clinical presentation *[Cases number]*	Topics discussed *(cases number)*	MDTB Outcomes
Choroidal Melanomas^a^*[10/12]*	Treatment planning *(9 cases)*	Therapeutic *Brachytherapy (4 cases), enucleation (one case), orbital exenteration (one case)*
		Referral *→ to Oncologists (3 cases) (due to systemic metastasis)*
	Discussion of first presentation *(one case)*	Diagnostic *FNAB biopsy required*
Choroidal Metastases *[2/12]*	Evaluation of histopathological report *(one case, patient with lung adenocarcinoma)*	Diagnostic *Orbital/Brain MRI planned*
	Treatment planning *(one case, patient with colon adenocarcinoma and refractory ocular pain)*	Therapeutic *external beam radiotherapy*

^a^5 out of 10 patients were already enucleated; among these, 4 out of 5 revealed an extra-scleral extension of melanoma, for which brachytherapy sessions (3 cases) or an orbital exenteration (one case) was scheduled; one case was referred to Oncologists due to new-onset hepatic metastasis

#### Main points

**Fig. 10:**
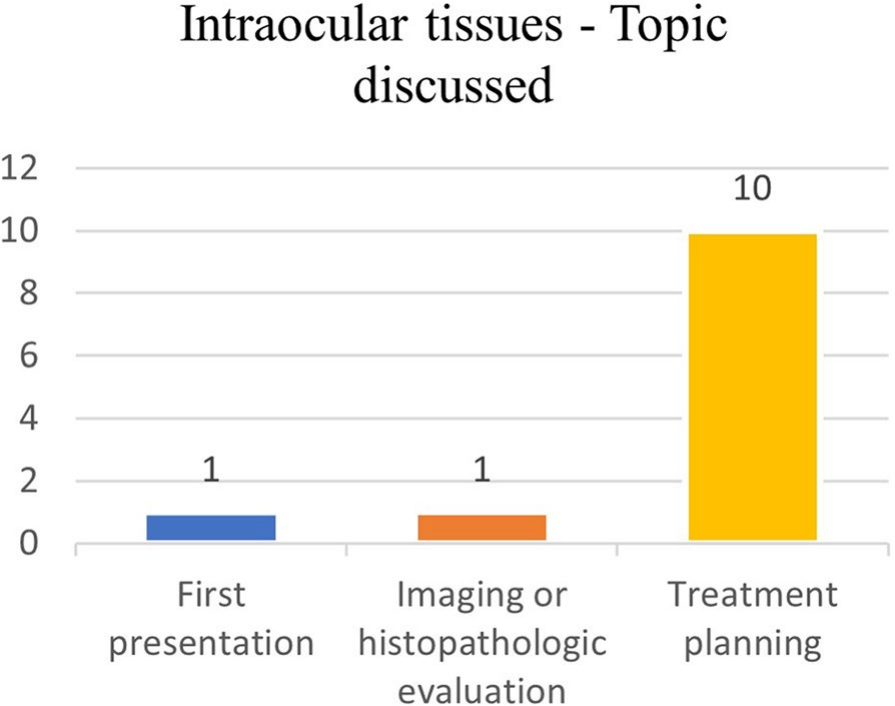
Intraocular tissues – Main topic discussed

Treatment planning was questioned for 10 out of 12 patients (83%); in one case a new clinical presentation was discussed, and in one case the histopathologic report was analyzed (Fig. 10).

#### Meeting outcomes

**Fig. 11:**
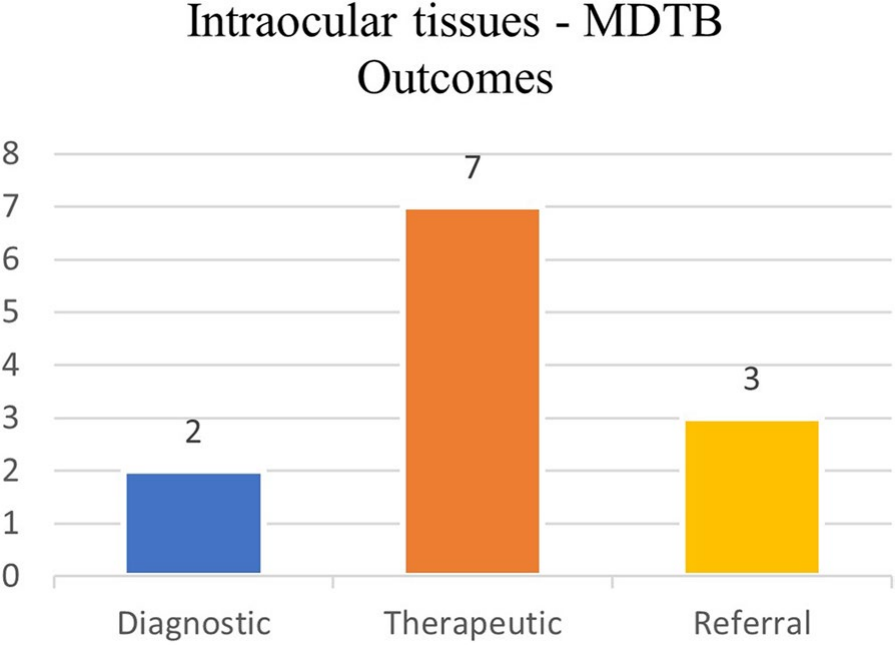
Intraocular tissues – Final decisions taken

A further diagnostic examination was required in 2 out of 12 patients (17%) via fine needle aspiration biopsy (FNAB) or an imaging study (orbital/brain RM). A therapeutic, surgical (eg: enucleation or exenteration) or radiotherapic (eg: brachytherapy sessions) treatment was recommended in 7 out of 12 patients (58%). Three patients (25%) with systemic disease (hepatic metastasis) were referred to Oncologists (Fig. 11).

## Discussion

In cancer management and treatment multidisciplinary meetings enable clinicians to discuss imaging results, increasing the likelihood of more precise or complete staging [5, 6].

The formulation of an optimal treatment plan may be facilitated by the discussion between different specialties regarding the advantages and disadvantages of various treatment approaches for specific patients [7]. Furthermore, as MacDermid et al. [8] suggested, MDTB meetings may facilitate patient access to oncological services: in their study, evaluating tumor board implementation in colorectal cancers, patients followed by a multidisciplinary team were more prone to receive adjuvant chemotherapy treatment resulting in a survival benefit.

Licitra et al. [4], in their experience in the treatment of Head and Neck cancer, reported that MDTMs could positively affect patients’ survival by improving accuracy in staging and adherence to clinical guidelines, providing cost-effective care and increasing overall satisfaction of patients. In addition, MDTB positively affects treatment decisions (eg: by changing tumor diagnosis, staging or treatment plan), reduces time to treatment and improves survival rate (particularly in early-stage cancers). Further investigations are ongoing to determine whether MDTBs are also cost-effective. For this, the MDTB approach is now considered the standard of care for management of patients with H&N cancers.

Within the field of Ocular Oncology, few data are available about the role of MDTB implementation. Shah et al. [9] reviewed the data collected over a 12-months period after the establishment of a weekly multidisciplinary team in a tertiary ocular oncology center. The main topics discussed, the rate of patient satisfaction and the changes in clinical diagnosis after discussion were reported in their letter. Clinical diagnosis was revised only in 5.2% of patients, and management plans were changed in 5%. According to the Authors, meetings were effective in defining a shared management of patients and had a positive psychological effect on the patients.

In our experience, multidisciplinary meetings provide the great advantage to gather different specialists with high expertise in their fields (Table 2). This allows an effective discussion of each clinical case within a reasonable duration – in our sample an average of 13 min – with saving in terms of time and costs. Despite an average of 13 days delay (corresponding to the “*time-to-presentation”* of cases from the clinic to meetings), and the mean waiting time for diagnostic ascertainments, therapeutic execution, or referral requests (27, 10 and 13 days respectively), the multidisciplinary meetings can be deemed an effective streamlining of ocular and periocular tumors management.

Regarding the MDTB outcomes, these were often (47 out of 86 cases, 55%) indications for treatment execution through different modalities (surgery, and/or chemotherapy, and/or radiotherapy). In 27 cases (31%) a further diagnostic work-up (CT/RMI imaging or incisional biopsy) was required. In 12 cases (14%), patients were referred to other specialists for medical or surgical treatment.

Clinical imaging (MRI or CT scans) evaluation was the main topic discussed in orbital tumors, while histopathologic reports were often analyzed in eyelid tumors cases.

Indications for referral and suggestions for diagnostic staging or restaging were prevalent in eyelid tumors group.

Ocular surface tumors group had the highest rate of discussions about treatment planning, resulting in therapeutic indications through surgical (eg: excisional biopsies) and/or radiotherapy (eg: brachytherapy) treatment; half of the patients among which treatment planning was discussed (10 out of 20) had already been previously treated and showed features of relapsing disease (eg: local recurrence or metastasis).

Discussions about intraocular tumors mainly concerned on treatment planning through multimodal approaches (enucleation, orbital exenteration or brachytherapy). Only particular cases of choroidal tumors with challenging management (eg: patients already enucleated with histological extra-scleral extension of melanomas) have been discussed during multidisciplinary meetings; patients with typical presentations have been treated according to standard protocols.

In literature it has been reported that multidisciplinary discussions can seldom (5.2%) lead to changes in clinical diagnosis [9]. In our sample, in two cases (2.3% out of 86) a requested revision of histopathological specimens led to a revised diagnosis of malignant orbital and ocular surface tumors, de facto changing a previously mismatched diagnosis and, therefore, the treatment plan.

In summary, the establishment of a monthly ocular oncology MDTB meeting in our center has been effective in defining the management of patients with ophthalmic cancers with the collaboration and support of various specialists.

Multidisciplinary meetings are increasingly adopted by oncologists among different specialties and, within the field of Ocular Oncology, they appear to be particularly helpful in cases with gray areas, when malignancies require a clinical management that spans among different specialties, or in patients requiring a second opinion of doubtful lesions. In these cases, open-ended discussions can stimulate the participation of the whole team toward reaching a shared treatment management avoiding the bias of a previously scheduled planning. A not measurable impact of MDTB on previous plans is the disadvantage of this approach. This study has several limitations: an analysis of advantages of ocular and periocular tumors meetings in terms of better prognosis, costs saving, and patients’ confidence improvement was not conducted. However, for most common cancers an evaluation of advantages in terms of better prognosis and survival rate is likewise not easy to determine [10], since the results should be compared to a valid control group in studies with a “no-MDTB” arm; a proposed “before and after” implementation [4] study design could partly by-pass this problem with the bias, however and luckily, that cancer assessment, diagnosis and treatments are rapidly developing.

## Conclusion

In conclusion, to the best of the authors’ knowledge, and except for Shah et al. [9] letter, this is the first report that describes the implementation and outcomes of a multidisciplinary approach for patients with ocular and periocular cancers, also providing suggestions about the team composition. From a diagnostic and therapeutic standpoint, in our experience this approach has revealed effective in both providing a holistic evaluation of oncologic patients - by sharing expertise of different team members - and in defining an integrated therapeutic management, also streamlining the redirection of patients among different specialists. Further studies are needed to investigate whether this method also translates into an increased survival rate and a better prognosis.

## Data Availability

The datasets generated and analyzed during the current study are available from the corresponding author on reasonable request.
